# Quantifying prescribed high dose opioids in the community and risk of overdose

**DOI:** 10.1186/s12889-021-11162-4

**Published:** 2021-06-24

**Authors:** Joe Schofield, Deborah Steven, Rebecca Foster, Catriona Matheson, Alexander Baldacchino, Andrew McAuley, Tessa Parkes

**Affiliations:** 1 Salvation Army Centre for Addiction Services and Research, Faculty of Social Sciences, Colin Bell Building, University of Stirling, Stirling, FK9 4LA Scotland; 2 Fife Pain Management Service, NHS Fife, Lynebank Hospital, Halbeath Road, Dunfermline, KY11 8JH Scotland; 3 Population and Behavioural Science Division, School of Medicine, University of St Andrews, North Haugh, St Andrews, KY16 9TF Scotland; 4 NHS Addiction Services, NHS Fife, NHS Fife, Lynebank Hospital, Halbeath Road, Dunfermline, KY11 8JH Scotland; 5 School of Health and Life Sciences, Glasgow Caledonian University, Cowcaddens Road, Glasgow, G4 0BA Scotland; 6 Health Protection Scotland, Public Health Scotland, 5 Cadogan Street, Glasgow, G2 6QE Scotland

**Keywords:** Prescription opioids, Overdose, Analgesics, Comorbidities, General practice, Polypharmacy

## Abstract

**Background:**

Opioid prescribing for a range of health issues is increasing globally. The risk of fatal and non-fatal overdose is increased among people prescribed strong opioids: in high doses in the context of polypharmacy (the use of multiple medications at the same time), especially with other sedatives; and among people with multiple morbidities including cardiorespiratory, hepatic and renal conditions. This study described and quantified the prescribing of strong opioids, comorbidities and other overdose risk factors among those prescribed strong opioids, and factors associated with high/very high opioid dosage in a regional health authority in Scotland as part of a wider service improvement exercise.

**Methods:**

Participating practices ran searches to identify patients prescribed strong opioids and their characteristics, polypharmacy, and other overdose risk factors. Data were anonymised before being analysed at practice and patient-level. Morphine Equivalent Doses were calculated for patients based on drug/dose information and classed as Low/Medium/High/Very High. Descriptive statistics were generated on the strong opioid patient population and overdose risk factors. The relationship between the prescribing of strong opioids and practice/patient-level factors was investigated using linear and logistic regression models.

**Results:**

Eighty-five percent (46/54) of GP practices participated. 12.4% (42,382/341,240) of individuals in participating practices were prescribed opioids and, of these, one third (14,079/42,382) were prescribed strong opioids. The most common comorbidities and overdose risk factors among strong opioid recipients were pain (67.2%), cardiovascular disease (43.2%), and mental health problems (39.3%). There was a positive significant relationship between level of social deprivation among practice caseload and level of strong opioid prescribing (*p* < 0.001). People prescribed strong opioids tended to be older (mean 59.7 years) and female (8638, 61.4%) and, among a subset of patients, age, gender and opioid drug class were significantly associated with prescribing of High/Very High doses.

**Conclusions:**

Our findings have identified a large population at potential risk of prescription opioid overdose. There is a need to explore pragmatic models of tailored interventions which may reduce the risk of overdose within this group and clinical practice may need to be tightened to minimise overdose risk for individuals prescribed high dose opioids.

**Supplementary Information:**

The online version contains supplementary material available at 10.1186/s12889-021-11162-4.

## Background

Scotland has one of the highest rates of opioid-related overdose mortality in Europe, with the rates of fatal overdose continuing to increase [1]. These overdoses mainly occur among users of illicit opioids such as heroin, or opioids prescribed for dependence such as methadone and buprenorphine [1]. However, individuals who are prescribed opioids for clinical reasons can also have increased Prescription Opioid Overdose Risk (POOR) [2, 3]. While deaths involving prescription opioids are not routinely reported as a discrete group in Scotland, of the 1264 drug-related deaths in Scotland in 2019, opiates/opioids were implicated in 1092 (86%) including prescribable opioids such as methadone, buprenorphine, and codeine/dihydrocodeine, reflecting a trend observed in recent years [1].

Individuals are prescribed opioids for acute pain, cancer pain and chronic non cancer pain (CNCP); for the management for dependence on illicit opioids; and for palliative care [4]. Opioids can effectively manage acute and cancer pain but there is limited evidence of their efficacy in CNCP [5], with studies finding variable quality evidence of short to medium-term analgesic benefits. Studies have highlighted an increased risk of harm directly related to opioid prescribing [6–8]. In the last 20 years, there have been considerable increases in the prescription of opioids for CNCP, predominantly in high-income countries including the USA, Canada and Australia. This increase is perceived to be caused by several interrelated systematic, political, economic, prescribing and governance developments and issues that syndemically created a large population of people prescribed opioids, with questionable benefits [9–11]. Furthermore, this may have contributed to increasing or persistent expectations by patients for the unqualified continuation of these prescriptions [12, 13]. It also reflects the cultural and social standpoints and practices of clinical, legal and regulatory communities which fluctuate over time, as documented in the narrative review by Rosenblum and colleagues [14]. Some countries, including the USA and Canada, have responded to these concerns through increased monitoring or regulation of these prescriptions, although these measures have been associated with some unwelcome, unintended consequences such as increased use of *illicit* opioids [15, 16, 17].

In Scotland the number of individuals prescribed opioids for CNCP increased by 66% between 2006 and 2016 [18]. However, within this broader trend, opioid prescribing is not evenly distributed across society; areas experiencing higher levels of socio-economic deprivation tend to experience higher rates of opioid prescribing, as evidenced in England [19, 20, 21] and Scotland, particularly in relation to the prescribing of strong opioids [22, 23].

While some literature may discuss two distinct groups of opioid users, clinically, the illicit opioid using and the CNCP populations share similarities [24]. A recent Scottish study of people over 35 years old with a drug problem found that 52% self-reported CNCP [25]. The CNCP population may overuse prescribed analgesia, be prescribed methadone, or buy illicit heroin and/or methadone; others become iatrogenically dependent due to using in excess of prescribed doses [26]. Australian studies have identified that those who were prescribed strong opioids or opioids on a longer-term basis for CNCP were those who experienced a range of physical and mental health challenges, as well as social disadvantage [26, 27]. Similarly, those who are prescribed opioids for pain related pathologies, palliative care, or illicit opioid dependency, often experience comorbidities contributing to POOR. Many cancer patients live with multiple comorbidities [28]. Those using illicit opioids often have co-occurring problems with substances including drugs and alcohol, as well as mental health problems [11, 29] and overdose risk is increased when these are present [11, 30, 31].

Additionally, polydrug use and polypharmacy practices are confounding factors that also merit consideration with national and international guidelines [32, 33] advising against the co-prescribing of these in combination with other sedatives, such as hypnotics and other anxiolytics (e.g. benzodiazepines), and gabapentinoids (gabapentin and pregabalin). Irrespective of the reasons for opioid prescribing, the risk of fatal and non-fatal overdose from co-occurring conditions may be increased in individuals where comorbidities compromise cardiorespiratory, renal and/or hepatic function [11].

Higher doses of opioids are also associated with increased overdose risk. The UK Faculty of Pain Medicine describes how the risk of harm increases substantially at doses of > 120 mg morphine equivalent dose (MED), with no additional clinical benefits at these doses [32]. Similarly, the United States’ Centers for Disease Control and Prevention (CDC) *Guidelines for Prescribing Opioids for Chronic Pain* notes “clinicians should use caution when prescribing opioids at any dosage, [and] should carefully reassess evidence of individual benefits and risks when increasing dosage to ≥50 morphine milligram equivalents (MME)/day, and should avoid increasing dose to ≥90 MME/day” [33].

In 2018, the Scottish Government and NHS Scotland published the *Quality Prescribing in Chronic Pain – a guide for improvement* guidance to encourage a quality improvement approach to the review of prescribing of key analgesic medicines in Primary Care including opioids and gabapentinoids [18]. These guidelines recommend that clinicians review patients co-prescribed analgesics and other potentially problematic drugs including gabapentinoids, and initiate conversations with patients about expectations surrounding what pharmacological treatment, including increases in doses, can realistically be offered in light of the evidence [18].

### Rationale

To develop appropriate, tailored responses to reduce POOR, it is essential to understand the population at risk. This includes the number of individuals prescribed opioids, their demographic characteristics and their clinical profiles, including comorbidities, polypharmacy and prescribed doses. Previous analysis of dispensed medications in Scotland identified factors associated with strong opioid prescribing stratified by sociodemographic variables [22]. This study develops the understanding of risk relating to comorbidities and concomitant prescribing.

The aims of the study were two-fold. Firstly, building on previous quality improvement work undertaken in the NHS Fife regional health authority (‘Health Board’) area of Scotland [18], to quantify and analyse the nature of current prescribing of strong opioids in community (‘primary’) healthcare by providing absolute numbers of patients in participating General Practice (GP) practices. Secondly, to identify cohorts of individuals who may be at risk of prescription opioid overdose, due to dosing schedules being higher than recommended; physical and/or mental health comorbidities; known adverse drug interactions and/or any combination of these factors.

This quality improvement exercise formed part of a wider study which also explored the acceptability of a bespoke take-home naloxone package for individuals prescribed opioids for CNCP via qualitative interviews.

## Methods

This study used data extracted from the National Health Service (NHS) primary care information systems to identify and describe a cohort of patients potentially in the POOR category. Data searches were undertaken both at practice and patient-level.

### Setting and scope

NHS Fife is an administrative and geographical Health Board serving a population of approximately 370,000 with 54 general practices grouped into 7 locality-based clusters. Potentially eligible patients were those registered at participating GP practices on 31/10/19 who were identified using the Prescribing Information System for Scotland which holds collated data on all prescriptions written in the country [34]. Patients were eligible for inclusion if they had been prescribed one or more strong opioids, alone or in combination with benzodiazepines, z-drugs, and/or gabapentinoids in the six months preceding this study. Based on the team’s clinical expertise and the British National Formulary (BNF) categorisation [35], strong opioids were defined as medications containing buprenorphine in either tablet or patch formulation, diamorphine, fentanyl, hydromorphone, methadone, morphine, oxycodone, pentazocine, pethidine, tapentadol, or tramadol.

### Procedures and data extraction

No formal ethical and/or research governance approvals were required as this study was undertaken as part of a quality improvement process. Primary Care Clinical Directors and Cluster Quality Leads gave approval for co-author DS to invite all NHS Fife practices to participate. Data collection was led by the Fife Pain Management Service Lead Pharmacist (DS). Coding was developed and emailed to participating pharmacy teams, enabling them to extract relevant prescribing data from their clinical information system via EMIS Web, a UK-wide clinical IT system that stores and enables the sharing of information about patients and practices across health-care settings [36].

A search strategy was devised for consistency across the practices [see Additional File 1] and searches were run by practice pharmacy teams between October and December 2019. Two searches were conducted. The first captured practice-level data on prescribing of strong opioids and occurrence of co-morbidities. POOR risk factors (polypharmacy and co-morbidities) were identified by searching on Read codes (version 2), a clinical coding system used in primary care in Scotland since the 1980s that can be mapped to ICD10 [37]. The second search captured patient characteristics (age, gender) and information from strong opioid prescriptions (drug, dose, date). All patients prescribed strong opioids were included as it was not possible to differentiate between prescribing for cancer pain and CNCP in the timescale available to conduct the project. Searches were devised to exclude multiple counting of a drug for the same patient. This ensured that, for example, monthly prescriptions for a specific drug were counted only once within the study period. Therefore, the data relate to the number of differing drug items, and not the total number of prescription items issued per patient prescribed during the study period, which would be considerably higher.

Returns were pseudonymised by replacing practice names with a numeric identifier. Practice returns were collated in an encrypted, password-protected Microsoft Excel document only accessible to members of the study team involved in data analysis. Each practice returned two files: firstly, practice-level data on the numbers of patients who received opioid prescriptions, concomitant prescribed high-risk medicines, or relevant comorbidities. Secondly, patient-level data including age, gender and details of prescribed strong opioid items including drug name, dose and date of issue. All participating practices consented for their pseudonymised data to be reported in aggregated cluster and health board levels. All but one practice consented for pseudonymised practice-level data to be reported; this practice’s data were included in aggregated summary statistics but excluded from practice-level reports. NHS Information Services Division Scotland provided data on practice population by deprivation quintiles as at the end of June 2019, using the Scottish Index of Multiple Deprivation (SIMD) definitions which quantifies local deprivation based on seven domains: income, employment, education, health, access to services, crime and housing [38].

### Analysis

Descriptive statistics and statistical models were applied using R version 4.0.0 [39] and the significance level for tests and models was set at 5%. For practice-level data, tables and summary statistics were generated to describe the number and proportion of practices participating and patients included overall and by cluster. The proportion of patients at participating practices prescribed strong opioids was summarised, as were the proportions prescribed any opioid and concurrent prescribing of strong opioids plus other central nervous system depressant drugs for comparison. Data on ‘Opioids excluding Tramadol’ was provided as there is a lack of clinical consensus whether to consider Tramadol as a weak or strong opioid. Overdose risk factors including polypharmacy and comorbidities were described. An unadjusted linear regression was developed to describe the relationship between the proportion of a practice’s caseload resident in SIMD 1 areas and the proportion of patients prescribed a strong opioid.

Patient-level data were recoded to calculate the maximum daily MEDs prescribed for each patient using conversion factors informed by the literature [40, 41] and available as an additional file [Additional file 2]. Opioid conversion factors are an approximate guide only because comprehensive data are lacking and there is significant inter-individual variation. The total MEDs were then categorised as low, medium, high or very high in line with guidance from the CDC and the Faculty of Pain Medicine [32, 33]  (Table 1). MEDs were not calculated for patients prescribed buprenorphine tablets as this preparation does not have a calculable MED under current guidelines due to complex, non-linear pharmacokinetics. Prescriptions were classed as concurrent if they had matching date of issue.

**Table 1:** Morphine Equivalent Dose (MED) category calculations

MED Dose Range (mg)	Category
0–50	Low (L)
51–90	Medium (M)
91–120	High (H)
> 120	Very High (VH)

Descriptive statistics present the distribution of age, gender, drugs prescribed, and items per patient. MEDs were summarised for patients on a methadone-containing regimen versus those not prescribed methadone as this drug had the highest median MEDs among our cohort due to the high morphine equivalence conversion factor. The proportion of patients where MEDs could not be calculated, and the reasons for this, were described.

Patient characteristics associated with prescribing of L/M MEDs versus H/VH were tested using an independent samples *t*-test to assess the relationship with age, and a chi squared test for the relationship with gender. Differences in the proportion of patients prescribed VH meds and the proportion with missing MEDs were compared for patients prescribed methadone versus those on methadone-free regimens using two-sample tests for equality of proportions.

The association between prescribed MEDs and potential risk factors (age, gender and opioid class) was investigated using logistic regression. A subset of 4003 patients was selected for this model, including those with data to support calculation of maximum MEDs (the dependent variable); who were prescribed one opioid only (to allow comparison across drug classes); who were prescribed a ‘high-volume opioid’ prescribed to more than 50 individuals in the sample; and where at least 10 patients were prescribed a H/VH dose: methadone (*n* = 272), fentanyl (242), morphine (2136), oxycodone (1353). Associations between predictive factors and the outcome were expressed as Odds Ratios (OR) and associated 95% confidence intervals (CI).

## Results

### Participation

Forty-six of the 54 invited GP practices in NHS Fife participated, a response rate of 85%. Of the eight non-participating practices, three declined and five were willing to participate but could either not run the search (*n* = 4) or were unable to provide all the required data (*n* = 1). The proportion of practices participating in each cluster ranged from 40% to 100%, with all but one achieving over 80% (Table 2). With a combined practice list size of 341,240, participating practices represented 88.4% of registered GP patients in NHS Fife. The median (Inter-Quartile Range, IQR) list size per practice was 7365 (3704) patients.

**Table 2:** Practice participation and list size by cluster

Cluster	Practice participation		Proportion participating	Combined practice list size
	No	Yes		
1	1	5	83%	34,676
2	0	10	100%	65,247
3	0	8	100%	41,006
4	1	6	86%	46,832
5	1	6	86%	59,693
6	2	9	82%	69,211
7	3	2	40%	24,575
*NHS Fife*	*8*	*46*	*85%*	*341,240*

### Practice-level data

#### Opioid prescribing

**Fig. 1:**
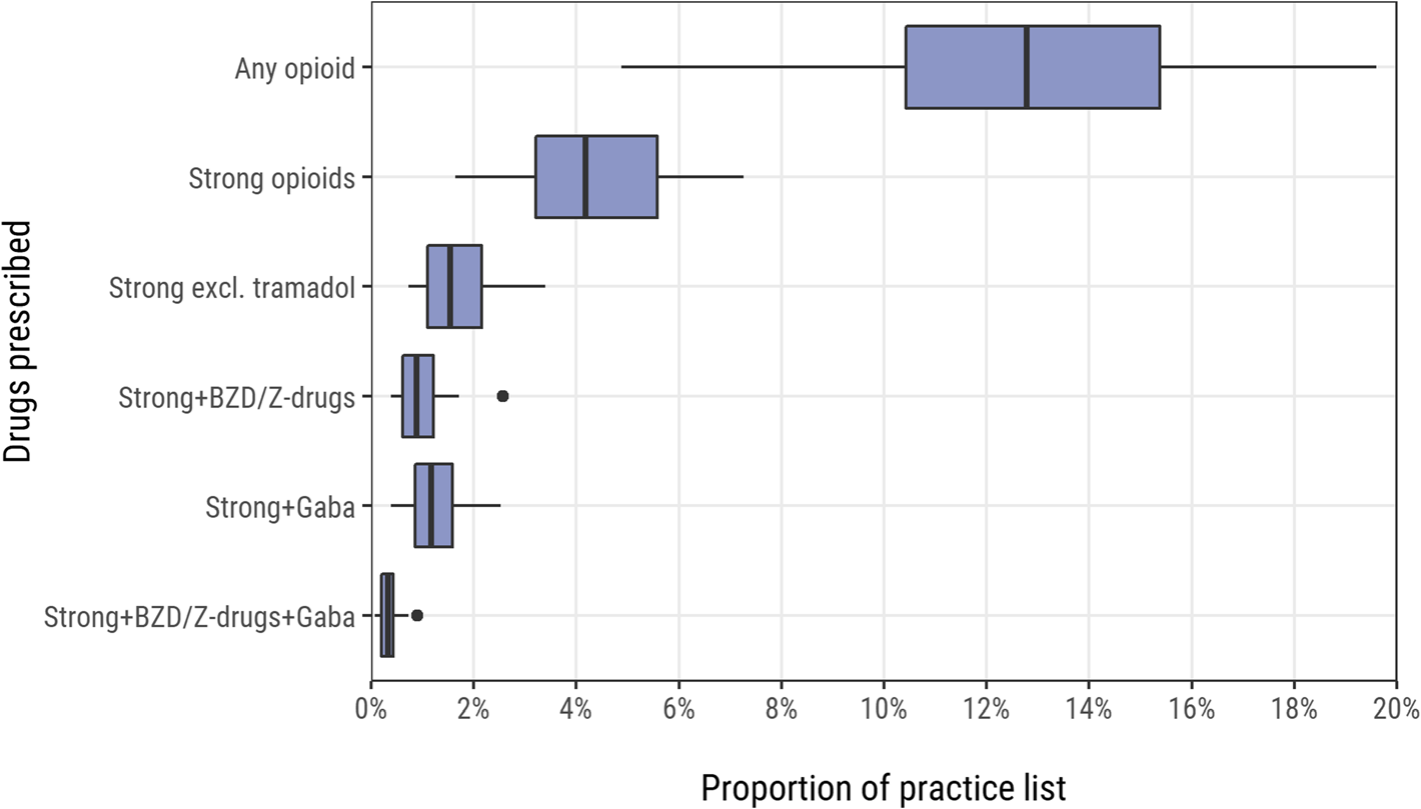
Opioid prescribing in the previous six months (per practice). BZD = benzodiazepines; Gaba = gabapentinoids

In the previous six months 42,382 (12.4%) of individuals were prescribed any opioid. Within these, 14,079 were prescribed a strong opioid, representing 4.1% of the overall caseload and 33.2% of patients who had been prescribed any opioid [see Supplementary Table 1, Additional File 3]. At practice-level, the median (IQR) number and proportion of patients prescribed a strong opioid were 262.5 (176.8) and 4.2% (2.4%) respectively (Fig. 1) [see Supplementary Table 2, Additional File 3].

#### Comorbidities and overdose risk factors

**Fig. 2:**
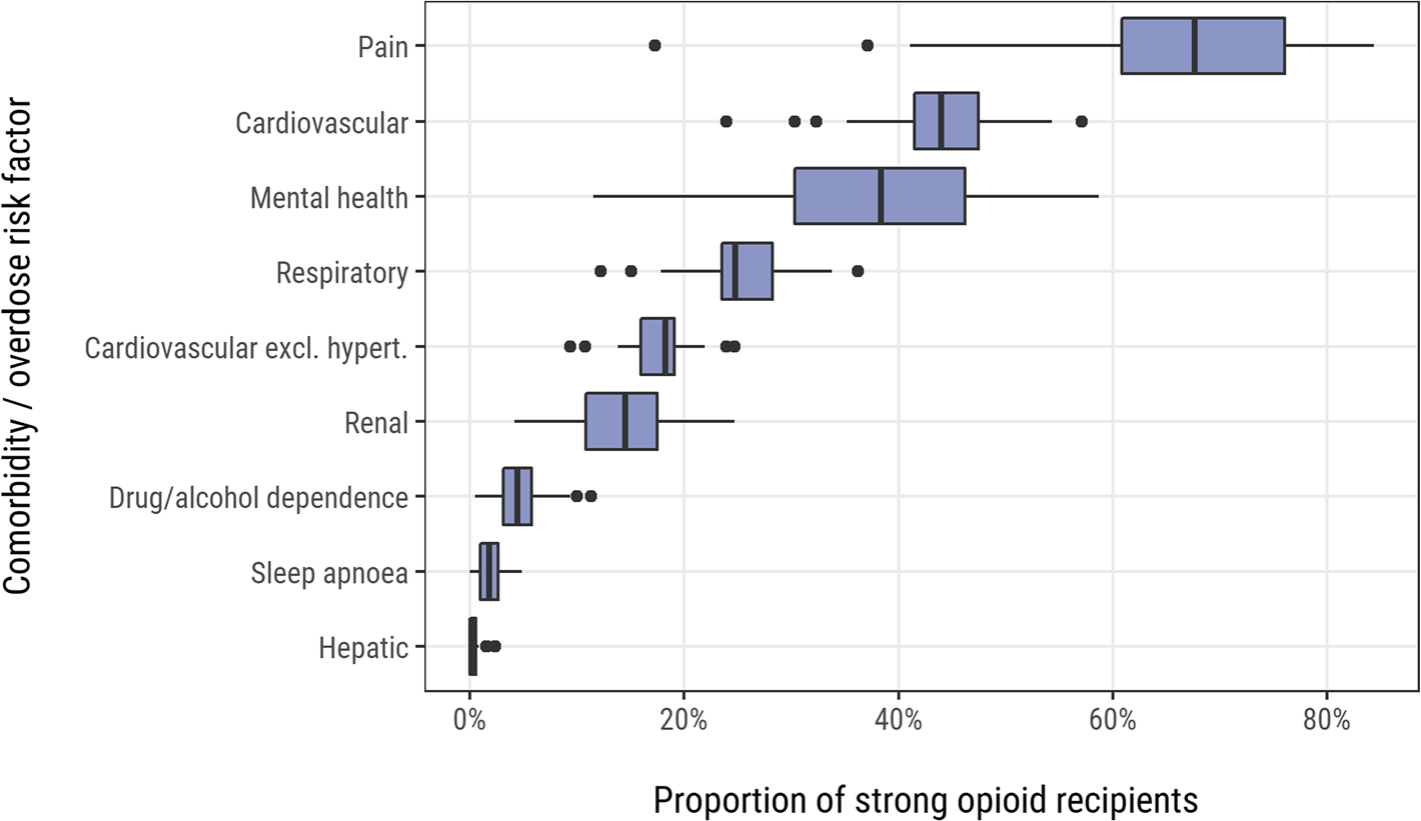
Recorded comorbidities and overdose risk factors among strong opioid recipients (practice-level). Excl. hypert. = excluding hypertension

Among the 14,079 patients who had been prescribed strong opioids, the most commonly reported comorbidities and overdose risk factors included pain (67.2%), cardiovascular disease (43.2%), mental health problems (39.3%) and respiratory disease (25.6%) [see Supplementary Table 3, Additional File 3], with evidence of variation in the distribution of these factors at practice-level (Fig. 2).

The proportion of the practice caseload resident in the most deprived (‘SIMD1’) areas was significantly associated with the proportion prescribed a strong opioid (*p* < 0.001) (Table 3). Model diagnostics indicated that one outlying practice, where 91% of the caseload lived in SIMD1 areas, exerted unusually high leverage in the model. The model remained significant (*p <* 0.001) when repeated with this practice excluded.

**Table 3:** Regression of proportion of caseload resident in SIMD 1 and proportion prescribed strong opioids

Coefficient (95% CI) *P* value	All practices (*N* = 46)	Excluding outlier practice (*N* = 45)
Constant	0.032 (0.027–0.037) *p <* 0.001	0.031 (0.026–0.036) *p <* 0.001
Proportion in SIMD 1	0.047 (0.032–0.062) *p <* 0.001	0.054 (0.037–0.072) *p <* 0.001
R^2^	0.465	0.478
Adj. R^2^	0.453	0.465
Model	*p <* 0.001	*p <* 0.001

### Patient-level data

**Fig. 3:**
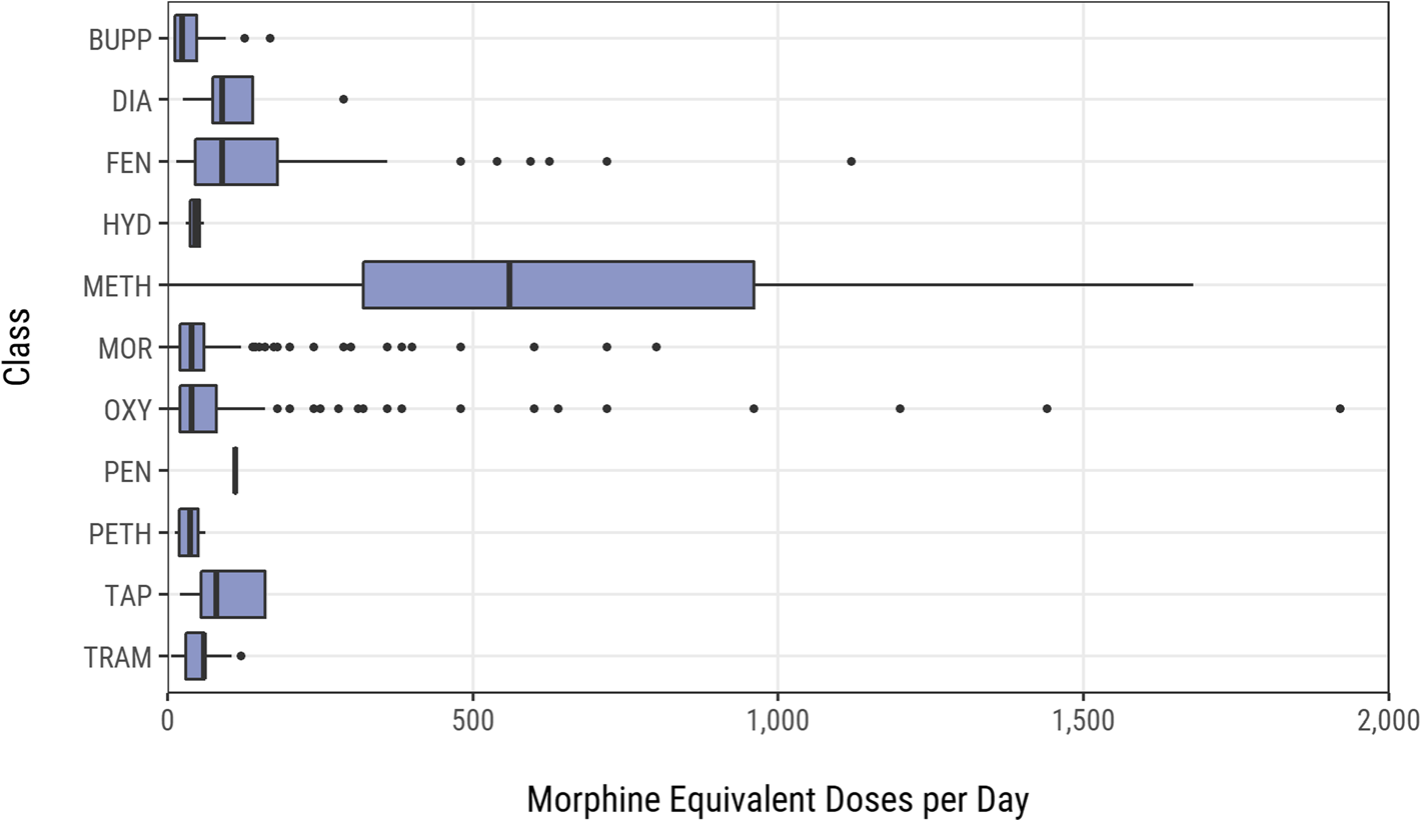
Prescribed MEDs by drug class (excluding buprenorphine tablets). BUPP = buprenorphine patches; DIA = diamorphine; FEN = fentanyl; HYD = hydrocodone; METH = methadone; MOR = morphine; OXY = oxycodone; PEN = pentazocine; PETH = pethidine; TAP = tapentadol; TRAM = tramadol

Practices provided data on 15,304 opioid items prescribed to 14,078 patients. Patient age ranged from 4 to 103 years with a mean (Standard Deviation, SD) of 59.7 (16.4) years. Most patients were female (8638, 61.4%), and the vast majority were prescribed one opioid (12,944, 91.9%). Eleven patients were aged less than 16 years with a median (IQR) age of 11 (7.5) years and a median (IQR) MED of 30 (41.8). The most commonly prescribed opioids were tramadol (9179 /15,304 items), morphine (3066/15,304), and oxycodone (1900/15,304). The range of MEDs per prescribed item was 0.4–1920 with a median (IQR) of 60 (30), and these values varied by drug class (Fig. 3) [see Supplementary Table 4, Additional File 3].

**Fig. 4:**
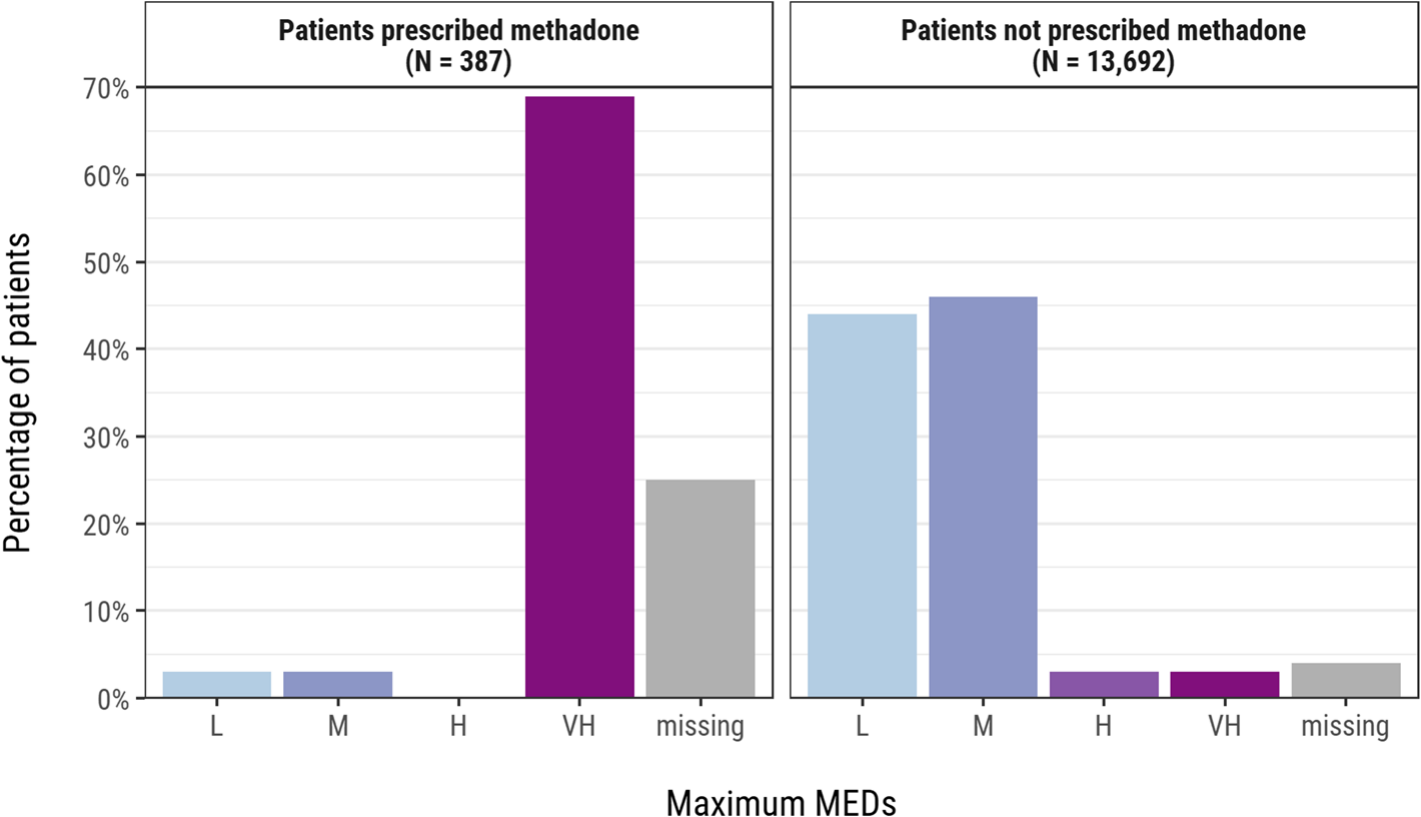
Maximum MEDs per patient by methadone prescribing status

There was a small but statistically significant difference in the age of patients prescribed L/M MEDs (mean 59.9, SD 16.4 years) versus H/VH MEDs (57.9, 17.0 years) (*t* (13,484) = 3.983, *p <* 0.001). There was also a statistically significant difference in the proportion of females who were prescribed H/VH MEDs (577, 6.9%) compared to males (522, 10.1%) (χ^2^ (1, *N* = 13,486) = 42.464, *p <* 0.001). Patients prescribed methadone-containing regimens were significantly more likely to have VH MEDs (265/387 vs. 433/13,692, *p <* 0.001), and to have missing MED information (95/387 vs. 497/13,692, *p <* 0.001) when compared to those not prescribed methadone (Fig. 4). [See Supplementary Tables 5 and 6, Additional File 3].

Multivariate analysis was conducted on a subset of 4003 patients, of whom 785 (19.61%) were prescribed H/VH MEDs. Age, gender, and drug class were all significantly associated with the likelihood of being prescribed H/VH MEDs (Table 4). After adjustment, male sex was associated with increased odds of H/VH MEDs (aOR 1.48, 95% CI 1.23–1.78, *p <* 0.001). Each additional year of a patient’s age was associated with a 1% reduction in the odds of receiving H/VH MEDs. Compared to methadone patients, those prescribed fentanyl (aOR 0.10, 95% CI 0.06–0.16), morphine (aOR 0.02, 95% CI 0.01–0.03) and oxycodone (aOR 0.03, 95% CI 0.02–0.05), had significantly reduced odds of being of being prescribed H/VH MEDs.

**Table 4:** Logistic regressions analysis of H/VH MED prescribing for 4003 patients

*Predictors*	Total, N	H/VH MEDs, n (% of N)	Unadjusted model			Adjusted model		
			*ORs*	*CI*	*P*	*aORs*	*CI*	*p*
(Intercept)			0.24	0.23–0.26	**< 0.001**	15.72	9.47–26.09	**< 0.001**
Age			0.97	0.96–0.97	**< 0.001**	0.98	0.98–0.99	**< 0.001**
Female (ref)	2485	378 (15.21)						
Male	1518	407 (26.81)	2.04	1.74–2.39	**< 0.001**	1.48	1.23–1.78	**< 0.001**
Methadone (ref)	272	248 (91.18)						
Fentanyl	242	86 (35.54)	0.05	0.03–0.09	**< 0.001**	0.10	0.06–0.16	**< 0.001**
Morphine	2136	238 (11.14)	0.01	0.01–0.02	**< 0.001**	0.02	0.01–0.03	**< 0.001**
Oxycodone	1353	213 (15.74)	0.02	0.01–0.03	**< 0.001**	0.03	0.02–0.05	**< 0.001**

ORs = Odds Ratios. aORs = Adjusted Odds Ratios; ref. = reference category; N = total number

#### Missing MEDs

It was not possible to calculate MEDs for 593 (3.87%) prescribed items, including 496 items where prescriptions did not include maximum daily dose information [see Supplementary Table 6, Additional File 3], and for 97 oral buprenorphine items due to the complex non-linear pharmacokinetics of this preparation. Maximum daily dose information was omitted from 23.8% (92/386) of all methadone items, 21.0% (4/19) of all pethidine items, and 33.3% (2/6) of all diamorphine items prescribed.

## Discussion

This study identified and quantified the size and nature of the population prescribed strong opioids in one NHS Health Board area in Scotland. 12.4% of individuals were prescribed opioids and, of these, one third were prescribed strong opioids. The population can be characterised as generally older patients with POOR comorbidities who were prescribed more than one analgesic medication. This group experienced a range of comorbidities with pain, cardiovascular/circulatory disease, and mental health issues being the most common.

Of particular concern regarding overdose risk are cardiovascular disease and respiratory disease [11] which were present in 43.2% and 25.6% patients respectively. Both compromise physiological resilience and further increase risk of overdose [11]. Mental health problems were frequently reported and are commonly associated with prescription opioid use, as well as the use of alcohol and other drugs [26, 27]. This study did not explore the *number* of comorbidities per patient; such analysis would be desirable in further research and enable more effective targeting of interventions. At practice-level, there was a positive relationship between levels of strong opioid prescribing and deprivation, in line with the referenced literature [19, 20, 21, 23]. Prescriptions lacking stated maximum daily dose information, for example those prescribed as ‘when required’, could further lead to inadvertent overdose by the patient or carer. Although methadone items were a small proportion of strong opioids prescribed among this cohort, patients on methadone-containing regimens were disproportionately exposed to very high MEDs, and methadone prescriptions were more likely to lack information on maximum daily dosing.

### Strengths and limitations of the study

There was a high level of participation (85%) in this study from GP practices. The reported data covers a high percentage of the NHS Fife population and should be broadly representative of prescribing activity and patient characteristics across the Health Board area. This study builds on previous analyses of opioid prescribing by volume and demographic characteristics, with the important addition of data on comorbidities and concomitant prescribing of high-risk medicines that could inform policy and practice developments across Scotland and beyond.

This study has several limitations. The level of Read coding across the participating GP practices was variable and so the number of patients with comorbidities that increase overdose risk may be underestimated. Comorbidity searches were conducted at population, rather than patient-level. This was compatible with the aims and parameters of this study. Data extraction relied on the goodwill of practice staff and pharmacy technicians and there was an associated trade-off between coverage and depth of data collection. As the analysis was undertaken on prescriptions generated at practice-level, and not those presented for dispensing via community pharmacy, the information must be regarded as a proxy measure of ‘intention to treat’ dosing data, rather than doses actually dispensed and/or consumed by patients. Prescriptions were classed as concurrent if they had the same start date and this conservative definition excluded other potentially overlapping prescriptions due to limitations in the data provided.

Given the decision to be inclusive of the entire POOR population, methadone and buprenorphine prescriptions were included, but these are less easily compared using MEDs. Methadone to morphine conversions are very different from other drug conversions and are dose-dependent, increasing exponentially as dose increases [41]. We opted to report methadone separately when calculating MED so as not to skew data. The criteria used to select patients for the analysis of prescribed MEDs and POOR risk factors resulted in just 4003 or 28.4% of the cohort being included in this analysis. Finally, the limitations of the searches meant it was not possible to differentiate prescribing for cancer pain, CNCP and other conditions.

In terms of clinical relevance, this study has quantified the potential size of the POOR population at health board, cluster and GP practice-levels, building on previous work in this area [18, 23]. Evidence of higher rates of strong opioid prescribing in areas of deprivation and the prevalence of comorbidities can help inform public health, prescribing, chronic disease management and other strategies to address possible overdose risk and reduce health inequalities. This study provides information and analysis in greater detail at practice level. It highlights issues around prescribed dosing through the absence of maximum recommended daily number of doses where prescriptions include ‘when required’ dosing information, or the absence of a prescribed dose altogether. In both cases this may increase risk of unintended overdose. It highlights the difficulty in assessing risk based on MED in some groups of patients e.g., those receiving buprenorphine tablets in opioid substitution therapy. It identifies groups of patients at potentially higher risk of opioid overdose events due to high dose opioid prescribing; use of concomitant prescribed medication which increase respiratory depression; and prescribing in patients with comorbidities that can increase opioid toxicity risk or are associated with higher risk of accidental or non-accidental overdose.

### Implications for policy and practice

Our results have several implications for opioid prescribing policy and practice to improve patient safety. Clinicians should routinely consider comorbidities that are associated with increased risk of overdose, polypharmacy, and total opioid MEDs when prescribing opioids. Prescribers should ensure there is no escalation of combined opioid doses above 90 mg MED in CNCP, in line with Scottish Intercollegiate Guidelines Network (‘SIGN’) 136 or equivalent international guidance [43]. Specific attention should be paid to MEDs and risk factors among patients receiving methadone-containing regimens.

Prescribers and dispensers (pharmacists) should provide patients with clear information on health conditions, behaviours and drug combinations that could place them at higher risk of overdose when taking opioids. Patients receiving opioids should also be offered regular review that includes assessment of the conditions for which opioids were indicated, concomitant risk factors, response to treatment, and discussion of alternative pain management and prescribing strategies. These recommendations depend on prescribers and dispensers being skilled and supported to deliver harm reduction conversations and interventions. In the context of large numbers of patients and limited clinical resources, a targeted approach could be considered which initially prioritises review of prescribed > 90 mg MEDs, and those with polypharmacy that includes opioids, gabapentinoids and hypnotics/anxiolytics. These recommendations correspond with recent findings from Campbell et al.’s [44] study which focused opioid-related harms for the CNCP population and encouraged a holistic assessment of the needs, risks and benefits for each individual, when addressing concerns of patient safety.increased use of illicit opioids

These findings demonstrate that additional clinical governance is required to reduce risk within this group. Greater leadership is needed on safer prescribing at national, regional, and local levels, with the importance of understanding prescribing in the context of an individual patient being a key part of this discussion. We recommend that other health authorities undertake similar audits and service improvement exercises, as well as interventions which may reduce the risk of overdose within this group. As outlined, there are a number of practical ways to reduce risk of inadvertent overdose, which also include the specification of maximum daily dosing in opioid prescriptions, minimising polypharmacy, especially with other CNS depressants, co-prescribing of naloxone, gradually tapering doses, and monitoring and responding to warning signs such as requests for early refills [45]. These need to be addressed or facilitated by prescribers or community pharmacists within a clear governance structure.

Finally, we acknowledge the potential for adverse consequences resulting from rapid reduction or withdrawal of prescriptions for people taking opioids that have been observed elsewhere, including the USA and Canada, such as a shift towards use of illicit opioids [15, 16, 17]. We also recognise that each country is unique and may have specific drivers for opioid prescribing which require tailored and patient-focussed responses. However, the findings from our study support others which highlight the risks of harm posed by prescribed opioids, and the need for decisive responses.

## Conclusions

This study described and quantified the population of primary care patients with POOR in one Scottish Health Board and described the characteristics of patients and their prescriptions that increase this risk. The study found a large cohort of patients prescribed strong opioids with increased POOR due to high doses, comorbidities, and polypharmacy. Other areas should undertake practice quality improvement exercises such as this, and develop staff training and patient-centred interventions, which may reduce the risk of overdose among this group.

## Supplementary Information

**Additional file 1.** Microsoft Word document, .docx. Additional File 1 – Search Strategy. Search strategy used in data extraction from practice records.

**Additional file 2.** Microsoft Word document, .docx. Additional File 2 - Morphine Equivalent Dose (MED) calculations. Data used to calculate MEDs for prescribed strong opioid doses.

**Additional file 3.** Microsoft Word document, .docx. Additional File 3 – Supplementary Tables. Supplementary tables.

## Data Availability

The data shared in this paper was obtained as part of an NHS service improvement exercise and did not include permissions for further sharing. Requests for data should be directed to the Corresponding Author.

## Abbreviations

aOR: Adjusted Odds Ratio
BNF: British National Formulary
BUPP: Buprenorphine Patches
BUPT: Buprenorphine Tablets
BZD: Benzodiazepine
CI: Confidence interval
CDC: Centers for Disease Control and Prevention
CNCP: Chronic Non-Cancer Pain
DIA: Diamorphine
DRD: Drug Related Death
FEN: Fentanyl
GP: General Practice
HYD: Hydromorphone
IQR: Inter-quartile Range
ISD: Information Services Division
L / M / H / VH: Low, Medium, High, Very High Morphine Equivalent Dose
MED: Morphine Equivalent Dose
METH: Methadone
MME: Morphine Milligram Equivalents
MOR: Morphine
NHS: National Health Service
OR: Odds Ratio
OXY: Oxycodone
PEN: Pentazocine
PETH: Pethidine
SD: Standard deviation
TAP: Tapentadol
TRAM: Tramadol
POOR: Prescription Opioid Overdose Risk
SIMD: Scottish Index of Multiple Deprivation, a deprivation rank for each of the 6505 data zones in Scotland
SIMD1: The most deprived quintile of Scottish data-zones
UK: United Kingdom
USA: United States of America
WHO: World Health Organisation
