# Supplementary material for: Quantifying prescribed high dose opioids in the community and risk of overdose
Source: BMC Public Health. 2021 Jun 24;21:1174. doi: 10.1186/s12889-021-11162-4 (PMC8223343; doi:10.1186/s12889-021-11162-4)
Supplement: Supplementary file 1 — Additional file 1. Microsoft Word document, .docx. Additional File 1 – Search Strategy. Search strategy used in data extraction from practice records. [file 12889_2021_11162_MOESM1_ESM.docx]

**Additional File 1: Search Strategy**

| **Search** | **Pain** | | **Alcohol & Drug Dependency** | | **Hepatic** | | **Mental Health** | | **Respiratory** | |  |
| --- | --- | --- | --- | --- | --- | --- | --- | --- | --- | --- | --- |
| **Read codes included** | Chronic pain  Chronic low back pain  Osteoarthritis  Generalised osteoarthritis  Hip osteoarthritis  Osteoarthritis NOS, Of hip  Osteoarthritis NOS  Osteoarthritis and allied disorders  Osteoarthritis NOS, Of hand  Generalised Osteoarthritis of multiple sites  Osteoarthritis of spine  Osteoarthritis NOS, Of pelvic region/thigh  Osteoarthritis of lumbar spine  Osteoarthritis of cervical spine  Primary Generalised Osteoarthritis  Rheumatoid Arthritis  Fibromyalgia  Sciatica  Multiple Sclerosis  Ankylosing Spondylitis  Unspecified polyarthropathy or arthritis  Cervicalgia- pain in neck  Cervical Spondylosis  Cervical disc degeneration  Other lumbar disc disorders  Spinal stenosis  Cervical spine stenosis  Migraine  Headache  Tension headache | | Misuse of drugs  Substance misuse of heroin  Misuse of cannabis unspecified  Drug dependence  Alcohol dependence syndrome  Alcohol intake above sensible limits | | Chronic active Hepatitis  Chronic hepatitis  Chronic viral hepatitis  Chronic viral hepatitis B  Chronic viral hepatitis unspecified  Acute hepatic Failure | | Anxiety states  Generalised Anxiety disorder  Anxiety state unspecified  Recurrent anxiety  [X] Depression NOS  Neurotic depression reactive type  Chronic depression  Post natal depression  On depression register  Agitated depression  Post Traumatic stress disorder  [X] Post Traumatic stress disorder  Mixed bipolar affective disorder  Mixed bipolar affective disorder, NOS  [X]Mixed bipolar affective disorder  [X]Other bipolar affective disorder  Organic bipolar affective disorder  Unspecified bipolar affective disorder  Paranoid Schizophrenia  Schizophrenia NOS  Schizophrenic disorders  [X]Schizophrenia  Chronic Schizophrenic  Paranoid Schizophrenia NOS  Chronic Paranoid Schizophrenia | | Practice Respiratory register | |  |
| **Search** | **Cardiovascular** | | **Cardiovascular Excl Hypertension** | | **Renal** | | **Sleep Apnoea** | |  | |  |
| **Read codes included** | Practice Cardiovascular register | | Practice Cardiovascular register excluding Hypertension | | Practice Chronic Kidney Disease register | | Sleep Apnoea | |  | |  |
| **Search** | **All opioids** | **Strong Opioids (CORE POOR group)** | | **Strong Opioids excluding tramadol** | | **Strong Opioids plus gabapentinoids** | | **Strong Opioids plus benzodiazepines & Z-Drugs** | | **Strong Opioids plus gabapentinoids plus benzodiazepines & Z-Drugs** | |
| **Search Terms for EMIS WEB** | Buprenorphine  Codeine Phosphate  Diamorphine  Dihydrocodeine  Dihydrocodeine Tartrate  Fentanyl  Fentanyl Citrate  Hydromorphone Hydrochloride  Morphine  Morphine Hydrochloride  Morphine Sulphate  Morphine Tartrate  Methadone Hydrochloride  Oxycodone hydrochloride  Oxycodone Pectinate  Pentazocine  Pentazocine Hydrochloride  Pentazocine Lactate  Pethidine Hydrochloride  Tramadol Hydrochloride  Tapentadol | Buprenorphine  Diamorphine  Fentanyl  Fentanyl Citrate  Hydromorphone Hydrochloride  Morphine  Morphine Hydrochloride  Morphine Sulphate  Morphine Tartrate  Methadone Hydrochloride  Oxycodone hydrochloride  Oxycodone Pectinate  Pentazocine  Pentazocine Hydrochloride  Pentazocine Lactate  Pethidine Hydrochloride  Tramadol Hydrochloride  Tapentadol | | Buprenorphine  Diamorphine  Fentanyl  Fentanyl Citrate  Hydromorphone Hydrochloride  Morphine  Morphine Hydrochloride  Morphine Sulphate  Morphine Tartrate  Methadone Hydrochloride  Oxycodone hydrochloride  Oxycodone Pectinate  Pentazocine  Pentazocine Hydrochloride  Pentazocine Lactate  Pethidine Hydrochloride  Tapentadol | | **(CORE POOR group)**  **Plus**  Gabapentin  Pregabalin | | **(CORE POOR group)**  **Plus**  Hypnotics & Anxiolytics | | **(CORE POOR group)**  **Plus**  Gabapentin  Pregabalin  **Plus**  Hypnotics & Anxiolytics | |
