# Supplementary material for: Quantifying prescribed high dose opioids in the community and risk of overdose
Source: BMC Public Health. 2021 Jun 24;21:1174. doi: 10.1186/s12889-021-11162-4 (PMC8223343; doi:10.1186/s12889-021-11162-4)
Supplement: Supplementary file 2 — Additional file 2. Microsoft Word document, .docx. Additional File 2 - Morphine Equivalent Dose (MED) calculations. Data used to calculate MEDs for prescribed strong opioid doses. [file 12889_2021_11162_MOESM2_ESM.docx]

**Additional File 2: Morphine Equivalent Dose (MED) calculations**

Opioid conversion factors are an approximate guide only because comprehensive data are lacking and there is significant inter-individual variation (1-5). In the time between undertaking the study searches and conducting the analysis and write-up, the UK Royal College of Anaesthetists updated their conversion factors for several drugs including oxycodone and fentanyl which reduced the MED load for patients who received these drugs. Conversion factors remains an area open to debate, with continued variability between references (1-5) and are an approximation only. The following table reflects conversion factors used in the study analysis.

| **Drug** | **Form or dose range** | **Oral Morphine Equivalence Conversion Factor or Morphine Equivalence(mg) used in study** |
| --- | --- | --- |
| Buprenorphine | Transdermal patch | 5microgram/hr buprenorphine patch = 12mg MED |
|  | Tablets | N/A due to nonlinear pharmacology |
| Diamorphine | Subcutaneous or intranasal | 3 |
| Fentanyl | Transdermal patch | 12microgram/hr fentanyl patch = 30mg MED |
| Hydromorphone |  | 7.5 |
| Methadone | 1-20 mg/day | 4 |
|  | 21-40 mg/day | 8 |
|  | 41-60 mg/day | 10 |
|  | ≥ 61-80 mg/day | 12 |
| Morphine | Oral | 1 |
|  | Subcutaneous | 2 |
| Oxycodone | Oral | 1.5 |
| Pentazocine |  | 0.37 |
| Pethidine |  | 0.125 |
| Tapentadol |  | 0.4 |
| Tramadol |  | 0.15 |

**References**

1. Faculty of Pain Medicine of the Royal College of Anaesthetists (2020) **Dose equivalents and changing opioids** <https://fpm.ac.uk/opioids-aware-structured-approach-opioid-prescribing/dose-equivalents-and-changing-opioids> [Accessed 06/01/21]
2. British National Formulary (no date) **Prescribing in Palliative Care** <https://bnf.nice.org.uk/guidance/prescribing-in-palliative-care.html> [Accessed 06/01/21]
3. Practical Pain Management (2018) **Opioid Calculator** <https://opioidcalculator.practicalpainmanagement.com/methods.php> [Accessed 06/01/21]
4. Centers for Disease Control and Prevention (no date) **Calculating total daily dose of opioids for safer dosage** <https://www.cdc.gov/drugoverdose/pdf/calculating_total_daily_dose-a.pdf> [Accessed 06/01/21]
5. NHS Scotland Scottish **Palliative Care Guidelines: Choosing and changing opioids** (2021) <https://www.palliativecareguidelines.scot.nhs.uk/guidelines/pain/choosing-and-changing-opioids.aspx> [Accessed 06/01/21]
