# Supplementary material for: Quantifying prescribed high dose opioids in the community and risk of overdose
Source: BMC Public Health. 2021 Jun 24;21:1174. doi: 10.1186/s12889-021-11162-4 (PMC8223343; doi:10.1186/s12889-021-11162-4)
Supplement: Supplementary file 3 — Additional file 3. Microsoft Word document, .docx. Additional File 3 – Supplementary Tables. Supplementary tables. [file 12889_2021_11162_MOESM3_ESM.docx]

**Additional File 3: Supplementary Tables**

# Supplementary Table 1: Opioid prescribing in the previous six months (overall)

| Patients in sample N = 341,240 | | | |
| --- | --- | --- | --- |
| **Opioids prescribed in past six months** | **Prescribing among caseload** | | **Proportion of those prescribed any opioid** |
|  | **Number (n)** | **Percent (n/N)** |  |
| Any opioid | 42,382 | 12.42% | 100.00% |
| **Strong opioids (core group)** | **14,079** | **4.13%** | **33.22%** |
| Strong excluding tramadol | 5,614 | 1.65% | 13.25% |
| Strong + benzodiazepines / Z-drugs | 3,075 | 0.90% | 7.26% |
| Strong + gabapentinoids | 3,965 | 1.16% | 9.36% |
| Strong + benzodiazepines / Z-drugs + gabapentinoids | 1,110 | 0.33% | 2.62% |

# Supplementary Table 2: Opioid prescribing in the previous six months (per practice)

| **Opioids prescribed in past six months** | **Number of patients per practice** | | |  | **Proportion of practice list** | | |
| --- | --- | --- | --- | --- | --- | --- | --- |
|  | **Range** | **Median** | **IQR** |  | **Range** | **Median** | **IQR** |
| Any opioid | 235 - 1779 | 860 | 477.5 |  | 4.9% - 19.6% | 12.8% | 5.0% |
| **Strong opioids (core group)** | **82 - 701** | **262.5** | **176.8** |  | **1.6% - 7.3%** | **4.2%** | **2.4%** |
| Strong excl. tramadol | 27 - 287 | 95.5 | 77.3 |  | 0.7% - 3.4% | 1.5% | 1.1% |
| Strong + BZD/Z-drugs | 11 - 161 | 61.5 | 42.2 |  | 0.4% - 2.6% | 0.9% | 0.6% |
| Strong + gabapentinoids | 24 - 227 | 68.5 | 55.2 |  | 0.4% - 2.5% | 1.2% | 0.7% |
| Strong + BZD/Z-drugs + gabapentinoids | 3 - 62 | 22.5 | 18.5 |  | 0.1% - 0.9% | 0.3% | 0.2% |

# Supplementary Table 3: Comorbidities and overdose risk factors among strong opioid recipients (practice level)

| **Comorbidity / overdose risk factor** | **Number of strong opioid recipients** | | | **Proportion of strong opioid recipients (%)** | | |
| --- | --- | --- | --- | --- | --- | --- |
|  | **Range** | **Median** | **IQR** | **Range** | **Median** | **IQR** |
| Pain | 45 - 511 | 161.5 | 148.8 | 17.3 - 84.3 | 67.6 | 15.1 |
| Cardiovascular / circulatory disease | 39 - 302 | 112.0 | 80.3 | 23.9 - 57.1 | 44.0 | 6.0 |
| Mental health | 26 - 343 | 110.0 | 101.8 | 11.5 - 58.7 | 38.4 | 15.9 |
| Respiratory disease | 10 - 201 | 67.5 | 40.7 | 12.2 - 36.2 | 24.8 | 4.7 |
| Cardiovascular / circulatory disease excluding hypertension | 14 - 117 | 47.5 | 34.2 | 9.4 - 24.7 | 18.2 | 3.2 |
| Chronic renal disease | 6 - 113 | 39.5 | 27.5 | 4.2 - 24.7 | 14.5 | 6.7 |
| Drug / alcohol dependency | 1 - 51 | 12.5 | 11.8 | 0.5 - 11.3 | 4.4 | 2.5 |
| Sleep apnoea | 0 - 34 | 4.5 | 6.8 | 0 - 4.9 | 1.8 | 1.7 |
| Hepatic disease | 0 - 13 | 0.5 | 2 | 0 - 2.4 | 0.1 | 0.6 |

# Supplementary Table 4: Prescribed Morphine Equivalent Doses (MEDs) by drug type

| **Class** | **Min** | **Q1** | **Median** | **Q3** | **Max** |
| --- | --- | --- | --- | --- | --- |
| Buprenorphine patches | 12.0 | 12.0 | 24.0 | 39.0 | 168.0 |
| Diamorphine | 25.2 | 73.8 | 90.0 | 139.5 | 288.0 |
| Fentanyl | 15.0 | 45.0 | 90.0 | 180.0 | 1,120.0 |
| Hydrocodone | 30.0 | 37.5 | 45.0 | 52.5 | 60.0 |
| Methadone | 0.4 | 320.0 | 560.0 | 960.0 | 1,680.0 |
| Morphine | 1.5 | 20.0 | 40.0 | 60.0 | 800.0 |
| Oxycodone | 2.0 | 20.0 | 40.0 | 80.0 | 1,920.0 |
| Pentazocine | 111.0 | 111.0 | 111.0 | 111.0 | 111.0 |
| Pethidine | 12.5 | 18.8 | 37.5 | 50.0 | 62.5 |
| Tapentadol | 20.0 | 55.0 | 80.0 | 160.0 | 160.0 |
| Tramadol | 5.8 | 30.0 | 60.0 | 60.0 | 120.0 |

# Supplementary Table 5: Maximum MEDs per patient by methadone prescribing status (excluding missing MED)

| **Maximum MEDs**  (category and dose range, excl. missing) | | **Patients prescribed methadone-containing regimens** | | **Patients prescribed methadone-free regimens** | |
| --- | --- | --- | --- | --- | --- |
| Low | 0-50 | 12 | 4.1% | 5,989 | 45.7% |
| Medium | 51-90 | 13 | 4.5% | 6,302 | 48.1% |
| High | 91-120 | 1 | 0.3% | 393 | 3.0% |
| Very High | >120 | 265 | 91.1% | 428 | 3.3% |
| **Total** |  | **291** | **100.0%** | **13112** | **100.0%** |

# Supplementary Table 6: Items with no maximum daily dose by drug type

| **Type** | **Number of items with no max. dose** | **Proportion of items with no max. dose** |  | **Items prescribed overall** | **No max. dose as proportion of items prescribed** |
| --- | --- | --- | --- | --- | --- |
| Buprenorphine patches | 1 | 0.20% |  | 153 | 0.65% |
| Diamorphine | 2 | 0.40% |  | 6 | 33.33% |
| Fentanyl | 2 | 0.40% |  | 483 | 0.41% |
| Methadone | 92 | 18.55% |  | 386 | 23.83% |
| Morphine | 166 | 33.47% |  | 3,066 | 5.41% |
| Oxycodone | 82 | 16.53% |  | 1,900 | 4.32% |
| Pethidine | 4 | 0.81% |  | 19 | 21.05% |
| Tramadol | 147 | 29.64% |  | 9,179 | 1.60% |
| **Total** | **496** | **100.00%** |  |  |  |

# Supplementary Table 7: Number of strong opioid items prescribed per patient, overall and concurrently

| **Items per patient** | **Overall** | | **Concurrent** | |
| --- | --- | --- | --- | --- |
| 1 | 12,944 | 91.94% | n/a | |
| 2 | 1,045 | 7.42% | 192 | 1.36% |
| 3 | 86 | 0.61% | 2 | 0.01% |
| 4 | 3 | 0.02% | 0 | 0.00% |

Please note, prescriptions were classed as *concurrent* if they had matching date of issue.

#
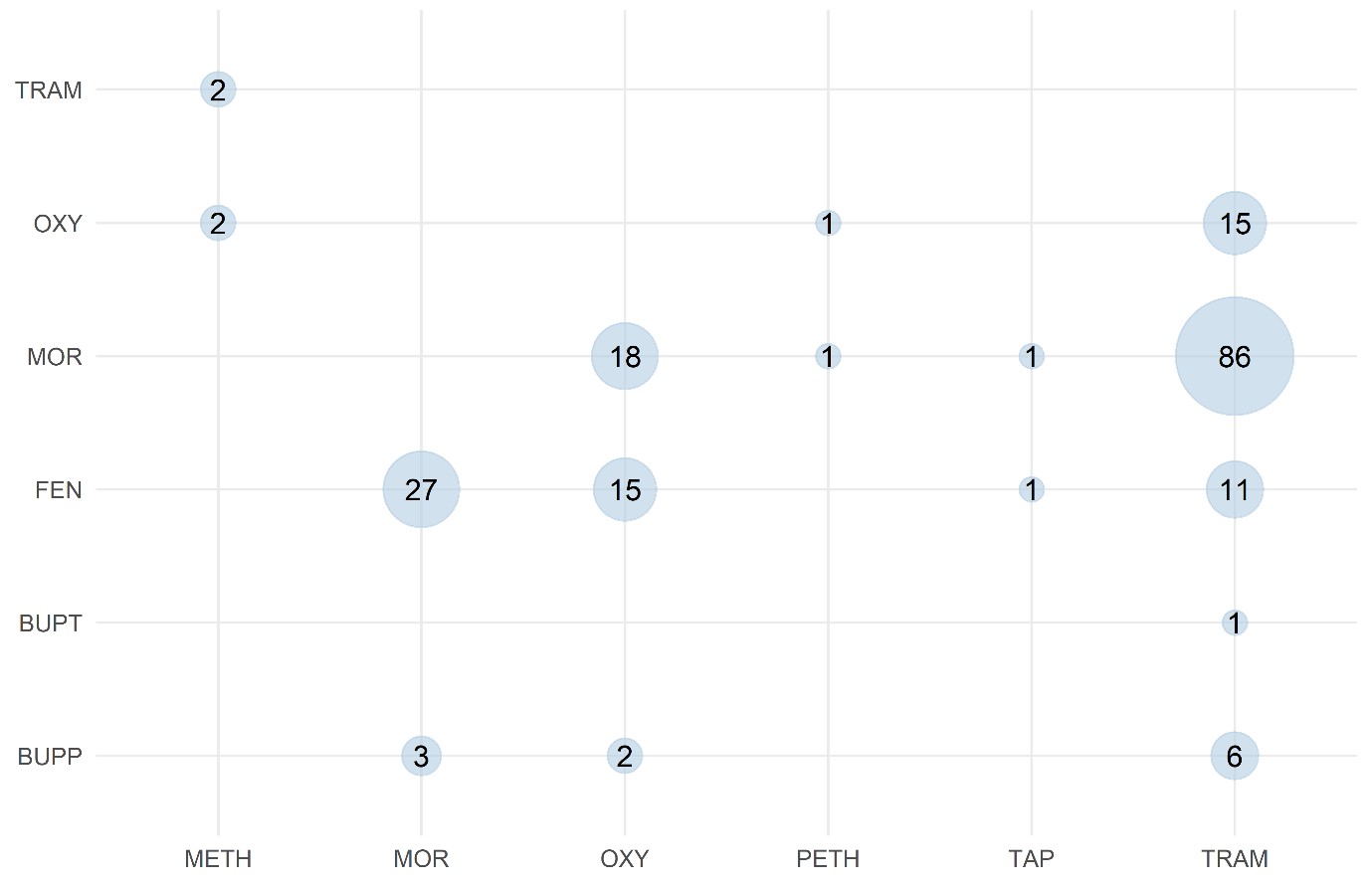
Supplementary Figure 1: Number of drug-drug combinations among patients with two concurrent prescriptions (N = 192)

BUPP = buprenorphine patch; BUPT = buprenorphine tablet; FEN = fentanyl; METH = methadone; MOR = morphine; OXY = oxycodone; PETH = pethidine; TAP = tapentadol; TRAM = tramadol.
